# Better 90 Minutes Late than Never: Differential Diagnosis on MRI Scanning in a Case of Hepatic Angiosarcoma

**DOI:** 10.3390/life14070823

**Published:** 2024-06-28

**Authors:** Teodora Anca Albu, Nicoleta Iacob

**Affiliations:** 1 Faculty of Physics, West University of Timisoara, 300223 Timisoara, Romania; 2 ScanExpert, 300627 Timisoara, Romania; 3 Miclaus Diagnostic Hub SRL, 307200 Timisoara, Romania

**Keywords:** angiosarcoma, MRI, gadobenatedimeglumine, liver, hepatobiliary phase

## Abstract

Primary hepatic angiosarcoma (PHA) is a rare liver malignancy with few studies describing its radiological characteristics. This article aims to assess the imaging features of each of the multiple delayed contrast-enhanced magnetic resonance imaging (MRI) scans, in addition to the conventional MRI protocol, in a patient with PHA. Standard MRI sequences and a liver protocol were used in the examination of a 71 year-old male with pathologically proven PHA after current imaging evaluation. In addition, the patient underwent transversal and coronal MRI T1-weighted scans at 10 min, 20 min and 90 min after intravenous (IV) administration of gadobenatedimeglumine (Gd-BOPTA). The PHA revealed a variable appearance on MRI, with classic imaging being insufficient in making a reliable diagnosis. Lesions have increased vascularity, which translates into increased IV contrast uptake in the MRI arterial phase, showing progressive and globular enhancement in the portal and parenchymatous phases. On delayed scans, at 10 min after IV administration, the lesions maintained no washout, but slightly began to washout at 20 min post-contrast. However, in the hepatobiliary phase (90 min post-contrast injection), on an MRI T1-weighted sequence, PHA lesions were hypointense, suggesting the absence of hepatocytes, thus indicating high-grade malignancy. This approach proved the conclusion that in a patient with PHA, an extra MRI T1-weighted scan at 90 min post-gadobenatedimeglumine injection can provide helpful information in differential diagnosis.

## 1. Introduction

Angiosarcoma represents a form of high-grade, aggressive malignant neoplasm, which consists of endothelial cells of vascular or lymphatic origin, presenting atypia, and can develop along pre-existing vascular sites, sinusoidal or cavernous areas. Moreover, it can also form clusters of blood vessels, solid masses or nodules. The radiological appearance can vary in accordance with tumor grading. Low-grade angiosarcomas are presented as a small, abundantly vascular, solid component with low-grade cytology. On the other hand, high-grade lesions are densely cellular and infiltrative with a high mitotic rate, displaying a varying amount of vascular formation that can appear focal [1,2]. Because of its endothelial origin, angiosarcomas can develop anywhere in the body and can occur at any age, although the most prevalent age range is between 60 and 71 years old, with a predisposition among the male population. Anatomically, it occurs most frequently in the head and neck, followed by the breast, with the liver being the fifth most common site of angiosarcomas [3]. 

Primary hepatic angiosarcoma (PHA) is a very rare (only 200 cases annually worldwide), but highly aggressive and invasive cancer with a poor prognosis. The most common factor is vinyl chloride monomer, followed by colloidal thorium dioxide, arsenic, radium diethylstilbestrol and urethane), although around 75% of PHA still has no known etiology [4]. PHA patients are prone to developing jaundice, ascites, weight loss and portal vein thrombosis, among other complications [5,6,7]. The majority of untreated patients die within 6 months—1 year. The reason for a late medical care approach from the patient could be the often silent clinical presentation, consisting of the rapid onset of non-specific symptoms: upper right abdominal pain, weight loss, fatigue and/or a slightly enlarged area in the right hypochondrium [8,9]. 

Radiological imaging can be conducive to diagnosis, although PHA is difficult to differentiate from other hepatic tumors (hepatocellular carcinoma being the most common) due to the hypervascular feature of PHA [10,11]. Ultrasonography shows multiple nodules or a single mass with variable echogenicity from necrosis and hemorrhage inside the lesion [12]. In computer tomography imaging, PHA appears hypointense with multiple patterns of contrast enhancement, becoming partly or completely isodense in comparison with the hepatic tissue [13]. MRI can also be used to characterize PHA as it can reflect the hypervascular and heterogeneous nature of the lesions [14]. Invasive biopsy can easily lead to a spontaneous hemorrhage because of PHA’s abundant vessel composition. 

However, imaging features of PHA are variable on all modalities described above, making it a mandatory aim to find new imagistic methods to aid the process of determining and characterizing PHA. Thus, diagnosing hepatic angiosarcoma remains a challenge as the current literature is limited and mostly derived from case reports or small cohort studies, mainly describing conventional CT and MRI [15,16,17,18].

This article provides an MRI view of hepatic angiosarcoma, summarizing the key imaging characteristics of this rare cancer and adding to the standard protocol the multiphasic delayed scans after IV contrast enhancement.

To our knowledge, no study has been conducted to determine the value of adding to the conventional abdominal MRI of multiple scans at 10, 20 and 90 min after intravenous (IV) administration of gadobenatedimeglumine (Gd-BOPTA) in a patient with primary hepatic angiosarcoma. This article aimed to assess the role of each multiphasic timing scan. 

Moreover, the present paper evaluates if this practice could have a superior diagnostic benefit compared to a mere classical MRI investigation.

## 2. Detailed Case Description

### 2.1. Clinical Presentation

This paper was conducted on the basis of a 71 year-old male subject’s case, who presented at the emergency department describing dyspnea and epigastric pain, had non-specific abdominal symptoms and had no history of carcinogen exposure. 

Laboratory analysis of blood samples revealed the following abnormal values: elevated alanine and aspartate transaminase, thrombocytopenia and anemia. The common hepatic tumoral marker, AFP, was negative. A creatinine analysis to assess renal function, prior to CT/MRI examination, was in the normal range (0.73–1.18 mg/dL). 

**Figure 1:**
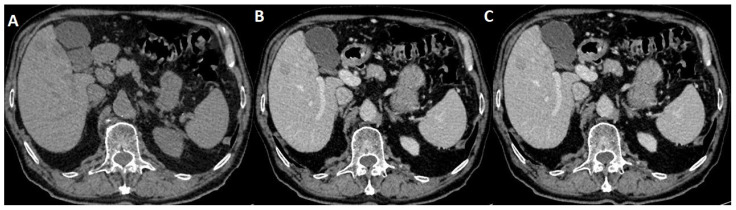
(**A**) Native transversal CT incidental findings on hepatic structure with multiple masses; (**B**) arterial iodate CT scanning showing hypoattenuating masses; (**C**) venous CT iodate appearance of nodular enhancement.

The patient then underwent an onsite thorax iodate CT, which on native scanning revealed an abnormal hepatic structure, so the CT operator performed an iodate arterial and venous thorax scanning, which also included the liver (Figure 1). 

A radiologic report of the thorax CT described incidental hepatic pathology consisting of hypoattenuating masses on both non-contrast and contrast-enhancedscans, and was not able to provide a certain diagnosis. After that, the patient was referred by the gastroenterology department to an imaging clinic for further abdominal MRI investigation.

### 2.2. Standard MRI Liver Protocol 

Abdominal imaging in the evaluation of liver pathologies has proven over time to be a valuable tool [19]. More precisely, the triple-phase liver protocol on both CT and MRI is currently part of the regular imaging assessment of focal liver lesions [20,21]. Scanning involves a dedicated late arterial phase, a portal venous phase and a delayed (parenchymatous) phase acquisition; during each imaging phase, scans are usually obtained in the transversal (tra) direction during a single 15–18 s breath-hold at deep inspiration. MRI was performed with a hepatocyte-specific contrast agent (gadobenatedimeglumine, Gb-BOPTA, trade name Multihance, Bracco). 

A conventional MRI protocol on 1.5 Tesla Siemens Sola, using an 8-channel abdominal phased array body coil, included: T2 WI HASTE (Half-fourier Single-shot Turbo spin-Echo) in the coronal (cor) and transversal (tra) planes (slice thickness 4 mm, gap 1.6 mm, matrix 198 × 256 mm, TR 1600, TE 94), T1 WI in and out phase tra (slice thickness 4 mm, gap 1.6 mm, TR 160, TE 2.38 and 4.76, respectively), T2 WI HASTE STIR (Short Tau Inversion Recovery) tra (slice thickness 4 mm, gap 1.6 mm, matrix 240 × 320 mm, TR 2000, TE 83), Diffusion WI tra with b values 50, 400, 800 s/mm^2^ (slice thickness 4 mm, gap 1.6 mm, matrix 108 × 134 mm, TR 2000, TE 51), T1 vibe FS (fat saturation) tra and cor, pre- and post-administration of contrast agent (slice thickness 3 mm, matrix 108 × 320 mm, TR 4.7, TE 2.38). 

On a T2-weighted imaging HASTE axial, hepatic angiosarcoma exhibits a heterogenous internal architecture that can mimic hepatocellular carcinoma.

Fat suppressed STIR T2 WI transversal images show multiple rounded lesions with a hyperintense signal, measuring a maximum of 5.14 cm in segment VII.

On craniocaudal T1 WI in- and out-of-phase, PHA displays heterogeneous areas of high signal reflecting a mixed tumor and hemorrhage.

**Figure 2:**
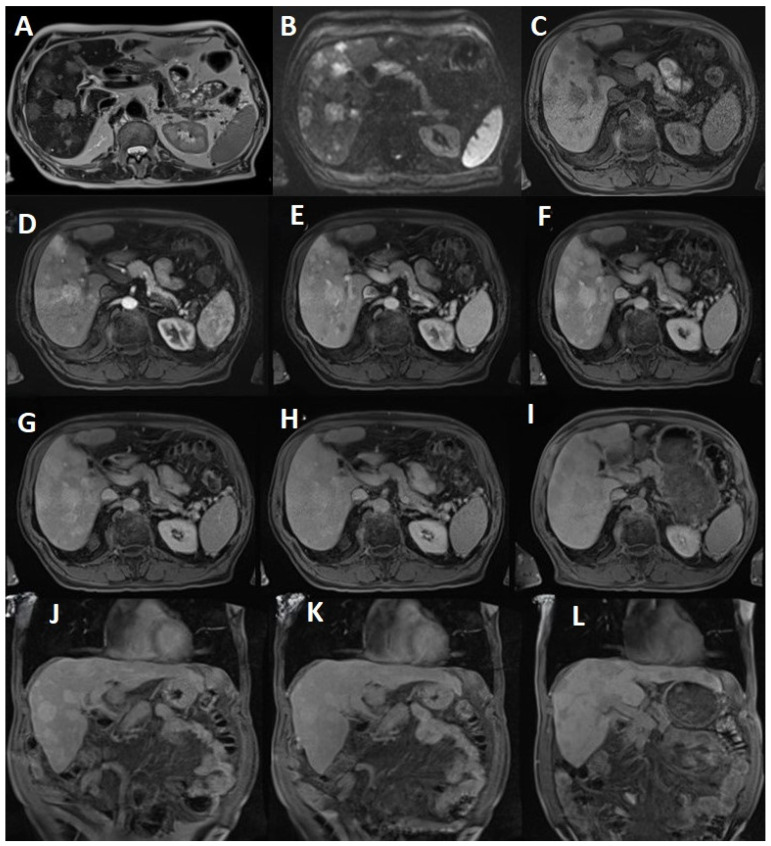
(**A**) T2 HASTE tra shows PHA lesions with a moderately intense signal, occupying almost 60% of the liver tissue. Increased signal intensity on T2 WI may demonstrate the presence of low flow vessels within the tumor; (**B**) Diffusion b value 800 suggests diffusion restriction; (**C**) T1 vibe FS tra pre-enhancement displays intermediate signal intensity with invasion of the surrounding tissue; (**D**) The arterial phase demonstrates high vascularity and perfusion that results in an increased rate of Gb-BOPTA uptake; (**E**) The venous portal phase exhibits progressive and globular enhancement; (**F**) The parenchymatous phase reveals heterogeneous enhancement with progressive filling; (**G**) The 10 min T1 vibe fs tra delayed phase produces no washout status; (**H**) The 20 min T1 vibe fs tra delayed phase merely exhibited the beginning of slight onset of washout; (**I**) On the 90 min T1 vibe fs tra delayed phase, the lesions became hypointense, suggesting the absence of hepatocytes; (**J**) The 10 min T1 vibe fs cor delayed phase; (**K**) The 20 min T1 vibe fs cor delayed phase; (**L**) The 90 min T1 vibe fs cor delayed phase.

Diffusion WI of b-value 800 s/mm^2^ shows corresponding hyperintensity of the lesion seen on T2. An apparent diffusion coefficient (ADC) map illustrates a low signal, confirming restriction and suggesting malignant composition (Figure 2A–C).

### 2.3. MRI Dynamic Three-Phasic Scanning

After routine axial images were obtained, one unenhanced (before IV injection of the contrast agent) fat-saturated T1 vibe in the transversal plane was first collected as a baseline. Then, Gb-BOPTA was injected into the elbow vein at 2.0 mL/s (the amount of contrast material was 0.1 mmol/kg body weight) using an automatic high-pressure injector (Ulrich GmbH&Co.KG, Buchbrunnenweg, Germany). Following injection, 20 mL saline was delivered at a rate of 2.0 mL/s to flush the injector and its accessory tube. Arterial, portal venous and delayed parenchyma T1 vibe tra scans were conducted.

On the arterial phase, contrast enhancement shows intense peripheral to center rim enhancement (potentially with central areas of necrosis), which acts in concordance with the histopathological hypervascular appearance.

The portalvenous phase post-contrast images reveal progressive rapid enhancement.

Through the parenchymatous phase, PHA lesions continuously maintain gadophile enhancement (Figure 2D–F).

### 2.4. Delayed Multiphase Scanning

Following the dynamic three phase protocol, the patient underwent extra scans using the same T1 vibe tra sequence at 10, 20 and 90 min. After a 10 min pause, the patient underwent the same transversal fat-saturated T1 WI vibe, which showed no washout status or significant changes among PHA lesions. Surprisingly, at 20 min, the scan showed a slight onset of washout. Moreover, contrary to the routine hepatospecific phase (120 min), the patient returned to the clinic at 90 min post-contrast administration, being examined with the same transversal fat-saturated T1 WI vibe. At this juncture, the lesions were hypocaptant, compared to the hepatic parenchyma, suggesting that the lesions did not have hepatocytes (Figure 2G–L). The 90-min delayed washout images (Figure 2I,L) are the most pertinent for identifying this pathology, while the other images are included to provide a basis for comparison.

These findings suggest that washout could appear earlier, even making it a characteristic feature for hepatic angiosarcoma (future research is needed on larger cohort); it is also logistically wise and important for the clinic schedule and more so for the patient (as it is quite difficult for a terminally ill patient to remain around for 2 h plus the MRI investigation). Until now, to our knowledge, there were no studies showing 10, 20 or 90 min delayed scan MRI imaging of hepatic angiosarcoma. 

The radiological report results’ differential diagnosis presentation consists of the following features: multiple nodular lesions (more than 20, measuring a maximum of 5.14 cm) occupying a significant surface of the liver tissue, heterogeneous areas of high signal on T2 and T2 fs, but hypointense on T1; they also present restriction on diffusion and all plead for a form of malignancy. Increased contrast uptake in the arterial MRI phase suggests a vascular component, negative for a rim-like or lollipop sign on the venous phase (differential diagnosis with hemangioendothelioma, which appears like a lollipop and is also situated predominantly on the peripheralsubcapsular) but has rapid and progressive enhancement in the portal, homogenous uptake on the tardive phase, without washout at10 min and a slight onset of washout at 20 min. On the hepatospecific phase, lesions are hypointense, suggesting the absence of normal hepatocytes.

Given the above imaging characteristics, a vascular malignant liver pathology, the appearance of multiple nodules, diffusion restriction with rapid uptake of contrast on the arterial phase, globular enhancement on the venous phase and homogenous uptake on the parenchymatous phase, washout starting at 20 min and completed at 90 min as a hepatocytic phase, hepatic angiosarcoma remans a potential diagnosis.

A biopsy was performed after the MRI scan and confirmed the imagistic diagnosis. Histopathology findings reveal an infiltrative vascular tumor with atypical endothelial cells displaying irregular hyperchromatic nuclei, while the immunohistochemical results were as follows: Hep-pal 1 (−), CD 34 (+), CD 31 (+), pattern p53* (+), proliferation index Ki-67 (10%+), which are all consistent with hepatic angiosarcoma.

## 3. Discussion

The present paper describes a rare case of PHA, which was incidentally first discovered on a thorax CT scan during a patient’s admission to the emergency department presenting with dyspnea and non-specific abdominal symptoms. Similar to other hepatic tumors, PHA is predisposed to inducing symptoms and complications, including ascites, hemorrhage and portal vein thrombosis [22,23]. A CT revealed hepatic masses suggesting malignancy, but for a more accurate diagnosis, the gastroenterology department requested an abdominal MRI.

In order to improve MRI evaluation of primary hepatic angiosarcoma, multiple scans after Gb-BOPTA IV administration were conducted on this patient and their added value was comprehensively analyzed. Several studies attest the importance of hepatospecific phase scanning at 1–3 h post-IV administration of gadobenatedimeglumine in liver pathologies, although none included primary hepatic angiosarcoma [24]. 

Aside from the conventional MRI liver protocol and dynamic three-phasic investigation (arterial, portalvenous, parenchymatous), scanning at 10 min, 20 min and 90 min post-contrast injection was included. From the latter additions, the 90 min time mark contributed the most to the differentiating PHA from other forms of liver tumor. Its incorporation to the standard evaluation could substantially improve the efficacy of diagnosis compared with that of routine examination alone, as PHA can mimic a variety of hepatic pathologies.

Because hepatic angiosarcoma is a rare hepatic tumor, limited articles and images were published in relation to imagistic diagnosis. The authors identified seven previous papers exploring MRI scans using gadobenatedimeglumine on patients with PHA. The cohort is limited in all studies due to the low prevalence of this pathology, hence the value of publishing individual reports for future research.

As stated in the previous papers [14,16,17,19,22,25,26,27] listed in Table 1, a complementary method should be used to ease the differential diagnosis. A solution appears to be late scanning after contrast agent administration, but further research is mandatory on a larger cohort as, so far, only one case report describes the added value of late scanning.

Table 1 includes the following features from the selected studies: sample size N (number of subjects), LCE (late contrast-enhanced) scanning and presumptive imaging diagnosis before the histopathological confirmation of PHA. The current case report was added to emphasize the novelty of the methodology utilized.

Summarizing the results listed in Table 1, late contrast-enhanced scanning holds significant promise as a future MRI characteristic for the more accurate diagnostics of PHA.

Concerning the multiphasic scanning, a number of measures were taken to acquire accurate data. First, a hepatocyte-specific contrast agent (GB-BOPTA) was utilized to assess the liver lesion, thus ensuring an optimal context for further delayed scanning. Secondly, the same sequence T1 vibe FS was used each time, performed at the end-inspiratory breath hold and verifying images for motion artifacts. Thirdly, an extra T1 vibe FS in the coronal plane was selected for an even better visualization of PHA lesions, for all the 10, 20 and 90 min delayed phases.

As a non-invasive and radiation-free examination method, MRI reveals the internal features of malignant lesions from multiple sequences, thus conferring abundant information. However, in relation to PHA, a routine examination proved to be insufficient, making it a necessity to search for new methods of sequence usage in order to help aid the process of differential diagnosis.

In this way, several studies state the usage of another hepatocyte-specific contrast agent (gadoxetate disodium, trademark Primovist) with similar results [28]. However, the high expense of this substance (compared to GB-BOPTA) caused a decreased intention among patients to undergo a gadoxetate disodium MRI examination, preferring the less expensive alternative.

Other studies, conducted on liver neoplasms in general, show the importance of scanning during the hepatospecific phase (1–3 h in theory), showing remarkable changes in the lesion appearance at 120 min post-contrast enhancement, and little to none at less than 120 min [29,30]. The issue with such a delayed time frame is that of the patient’s comfort and whole day’s schedule. In this way, the present study attempted to decrease the timing of the late scanning to a minimum but optimal number of minutes. In order to do so, it is mandatory to evaluate the quality of the hepatobiliary phase. If the hepatic parenchyma is hyperintense relative to the blood pool, the phase can be considered adequate. Results concluded that 90 min after IV contrast administration of GB-BOPTA proved to be sufficient in PHA.

Building upon the findings of this study, future research is needed on a larger cohort (which is an important limitation in the present paper) to assess if the 90 min delay time can also be decreased even less because at the 20 min mark, PHA lesions began to washout. 

## 4. Conclusions

Primary hepatic angiosarcoma is a rare condition, with a prevalence of just 200 cases annually worldwide; the life expectancy from first symptoms to death is 5 months, thus having enough patients for systematic research in a single center is difficult to achieve due to the low prevalence. Hepatic angiosarcoma is an absolute contraindication for liver transplantation. Chemotherapy options, including paclitaxel, gemcitabine/docetaxel, alkylating agents and anthracyclines, have not demonstrated a clear survival benefit for patients with this condition [31].

In the case of our patient, the lesions were deemed unresectable at the time of diagnosis. The patient underwent a CT scan, followed by a gastroenterology consultation, an abdominal MRI and a biopsy, which confirmed the diagnosis of primary hepatic angiosarcoma (PHA). Unfortunately, the entire diagnostic process took approximately 3 months. By the time everything was in place and the patient was scheduled for chemotherapy, he died of liver failure, not benefiting from any form of therapy.

By publishing this case report, the authors hope to inspire professional curiosity among other radiologists. In turn, these radiologists can perform similar types of scanning and publish additional images. If enough individual reports become available, a meta-analysis study can be conducted, increasing the likelihood of achieving statistical significance, ultimately contributing to better patient outcomes and more targeted treatment plans.

Hepatic angiosarcoma exhibits an ample spectrum of radiological appearances that reflect its diverse pathologic characteristics. Neither CT scanning with iodate contrast agent nor standard abdominal MRI can solely diagnose PHA. A classic MRI liver protocol augmented with quadruple-phasic scanning (arterial, venous, parenchymal and at 90 min post-IV administration of Gb-BOPTA) provide improved support in acquiring a differential diagnosis.

## Figures and Tables

**Table 1:** List of existing literatureand main characteristics of each paper.

Author/s	N	LCE Scanning	Presumptive Imaging Diagnostic
Current Case Report	1	10, 20, 90 min	PHA
Koyama T., et al. [14]	5	no	Hepatocellular carcinoma
Pickhardt P., et al. [16]	7	no	Cavernous hemangioma
Thapar S., et al. [17]	1	120 min	PHA
Zhang Y., et al. [19]	1	no	Diffuse angiogenic tumor
Flabouris K., et al. [22]	1	no	Peliosis hepatis or Hepatic epithelioid hemangioendothelioma
Heo SH, et al. [25]	1	no	Hemangioma
Wang J., Sun LT [26]	1	no	Malignant vascular tumor/features of infectious lesions
Xuwei W., et al. [27]	1	no	Multifocal hepatic hemangioma

## Data Availability

The data presented in this study are available on request from the corresponding author. The data are not publicly available due to confidentiality issues.
